# Investigation of a Family Cluster of Human Infections With Highly Pathogenic Avian Influenza A(H5N1) Virus, Clade 2.3.2.1e, in Cambodia, February 2023

**DOI:** 10.1111/irv.70231

**Published:** 2026-02-05

**Authors:** Savuth Chin, Chansovannara Soputhy, Heng Seng, Sokly Mom, Borann Sar, Alyssa Finlay, Kathrine R. Tan, Philip L. Gould, Jurre Y. Siegers, Erik A. Karlsson, Sonja J. Olsen, Timothy M. Uyeki, William W. Davis, Darapheak Chau, Sovann Ly

**Affiliations:** ^1^ Molecular and Virology Laboratory Unit National Institute of Public Health, Ministry of Health Phnom Penh Cambodia; ^2^ Communicable Diseases Control Department Ministry of Health Phnom Penh Cambodia; ^3^ Influenza Program, Communicable Diseases Control Department Ministry of Health Phnom Penh Cambodia; ^4^ Global Health Protection Division US CDC Phnom Penh Cambodia; ^5^ Influenza Division US CDC Atlanta Georgia USA; ^6^ Regional Influenza Program US CDC Hanoi Vietnam; ^7^ Virology Unit Institut Pasteur du Cambodge Phnom Penh Cambodia; ^8^ Regional Influenza Program US CDC Bangkok Thailand; ^9^ National Institute of Public Health, Ministry of Health Phnom Penh Cambodia

## Abstract

In February 2023, an 11‐year‐old girl in Cambodia developed severe respiratory symptoms and died of pneumonia and respiratory failure after testing positive for influenza A(H5). Contract tracing and testing identified her father as positive for influenza A(H5). Investigations revealed both were likely exposed to the same sick and dead poultry. Control measures included culling sick poultry and providing health education in the community on handling infected birds. Highly pathogenic avian influenza A(H5N1) viruses continue to pose public health risks in Cambodia.

## 1 Introduction

During 2005 through 2022, 56 human infections with highly pathogenic avian influenza (HPAI) A(H5N1) virus were reported in Cambodia. HPAI A(H5N1) viruses are endemic among poultry in Southeast Asia, including clades 2.3.2.1e (formerly classified as A(H5) clade 2.3.2.1c) and 2.3.4.4b [1, 2, 3, 4]. Clusters of influenza A(H5N1) cases in family members, which may indicate human‐to‐human transmission, are rare but have been documented [5, 6].

On February 14, 2023, an 11‐year‐old girl from Rumlech commune, Sithor Kandal District, Prey Veng Province, Cambodia, developed cough and fever. She was evaluated as an outpatient by a village nurse and clinicians at two private clinics and received antibiotics and antipyretics, laboratory tests, and intravenous fluids during February 16–20. On February 21, she was brought to the National Pediatric Hospital in Phnom Penh for worsening respiratory distress and was admitted to the intensive care unit. A chest X‐ray revealed bilateral pneumonia with diffuse bilateral alveolar opacities and left lung pleural effusion, and the patient was intubated for respiratory failure. Empiric oseltamivir treatment was started for suspected A(H5N1). Testing of upper respiratory tract specimens was negative for SARS‐CoV‐2 by rapid antigen test. The patient died on February 22. Because the hospital participates in surveillance for severe acute respiratory infection (SARI), nasopharyngeal (NP) and oropharyngeal (OP) swabs were collected just prior to the patient's death; these tested positive for influenza A(H5). Contact tracing and testing revealed that her father was also positive for influenza A(H5). This report describes the investigations and findings.

## 2 Methods

Staff from Cambodia public health, animal health, and environmental health agencies comprised rapid response teams; agencies included: Cambodia Communicable Diseases Control Department (C‐CDC), Ministry of Health (MoH) Field Epidemiology Training Program (FETP), National Institute of Public Health (NIPH), National Animal Health and Production Research Institute (NAPHRI), General Directorate of Animal Health and Production (GDHAP), and Ministry of Agriculture, Forestry and Fisheries. Objectives of the investigations were to identify additional A(H5) cases, determine exposure routes and modes of transmission, and implement control measures. Investigators interviewed family members, including the second case‐patient, and teachers to assess dates of illness onset, to identify potentially exposed persons. They interviewed the head of each household in the village to identify respiratory illness in the community, and a mobile clinic was set up in the village for influenza testing. Animal health officials interviewed village leaders about sick and dead poultry in the area, and they sampled poultry and the environment in areas where poultry were sick or died for influenza testing.

A case was defined as a person in Sithor Kandal District with laboratory‐confirmed influenza A(H5) virus detected in a respiratory specimen by real‐time reverse transcription polymerase chain reaction (rRT‐PCR). A suspected A(H5) case was defined as a close contact of a case or anyone in Rumlech Commune presenting with fever or cough from February 23 (when the investigation and active surveillance began) to March 10. A close contact was defined as anyone who was within 1 m of a confirmed case for at least 15 min from 1 day before symptom onset in the case and ending with isolation in the hospital or burial after death of the case. Close contacts were interviewed about type and duration of exposure and any personal protective equipment (PPE) worn; they were offered oseltamivir post‐exposure prophylaxis and monitored daily for 10 days after their last contact with a case and asked to report presence of fever (temperature of ≥ 100°F [37.8°C]) or feeling feverish/chills; cough; sore throat; difficulty breathing/shortness of breath; eye tearing, redness, or irritation; headaches; runny or stuffy nose; muscle or body aches; diarrhea.

Combined NP/OP swabs were collected from symptomatic and asymptomatic close contacts and suspected cases, stored in viral transport medium in cold boxes, and sent to the Virology unit of the National Institute of Public Health laboratories and the Institut Pasteur, both in Phnom Penh. Specimens were tested with rtRT‐PCR for influenza A and B viruses and SARS CoV‐2, and if positive for influenza A virus, they were tested with CDC subtyping kits for H1, H3, H5a, H5b, and N1 [7]. A(H5)‐positive specimens were sequenced using the Illumina MiSeq platform, and sequences were uploaded to GISAID. Viruses from both cases were isolated in viral culture using embryonated eggs.

Serum specimens were collected from 12 close contacts on February 23 and from nine of these again on March 17. Specimens were analyzed at Institut Pasteur by hemagglutination inhibition (HAI) assay to assess antibody reactivity against the isolated virus. The virus isolated from Case 2 A/Cambodia/2302009/2023 was propagated in embryonated chicken eggs, inactivated, and used as the HAI assay antigen. All sera were treated with receptor‐destroying enzyme (RDE) prior to testing, and chicken red blood cells (RBCs) were used for hemagglutination. Control ferret antiserum raised against A/Duck/Vietnam/NCVD‐584/2012 produced a homologous HAI titer of 1:640, confirming antigenic reactivity of the isolate with standard reference reagents.

This activity was reviewed by CDC, deemed not to be research, and was conducted consistent with applicable federal law and CDC policy.^1^

## 3 Results

Rumleach Commune comprises 1952 people in 375 households. Contact tracing and collection of upper respiratory specimens for influenza testing was initiated on February 23, including classmates and attendees of Case 1's funeral. On February 24, Case 1's father (Case 2) tested positive for A(H5); cycle threshold (Ct) value was 38.44 for H5a and 36.98 for H5b. Case 2 was a 50‐year‐old male farmer who lived in a house < 5‐min walk from the house where Case 1 lived. He developed a mild sore throat and cough on February 14; he took medication from the local pharmacy and recovered. He was asymptomatic on February 24 when he was isolated at the referral hospital, started on oseltamivir treatment, and placed under observation. He was discharged home on February 28 after NP/OP swabs collected on two consecutive days tested negative.

Viruses isolated from Cases 1 and 2 were identified as influenza A(H5N1) clade 2.3.2.1e in the Virology Unit at the Institut Pasteur du Cambodge; these were similar genetically to A(H5N1) viruses isolated from poultry in Cambodia in 2022 and 2023 [4]. The viral genomes obtained from Case 1^2^ and Case 2^3^ were nearly identical across all eight gene segments, differing by only a single nucleotide substitution in the PB1 gene (T1793C; L598P). This corresponds to 99.96% nucleotide identity across the PB1 segment. All other gene segments were completely identical. Full genome data for both viruses are publicly available on the GISAID EpiFlu database under the accession numbers listed in the footnotes. Paired serum specimens collected from Case 2, as well as eight other close contacts (including two who were symptomatic), were all seronegative by HAI assay.

Contact tracing of both cases identified 45 close contacts, including 24 healthcare workers (HCWs) and 18 people who attended the funeral, including Case 2; two reported fever, cough, or sore throat over the 14‐day period following exposure. Enhanced surveillance identified an additional eight people in the village who had fever and cough. The HCW who conducted the initial 30‐min consultation and examination did not wear any PPE; all others (including those who performed the intubation) wore at least surgical facemasks and cleaned their hands with alcohol handrub (Table S1). Of NP/OP swabs collected from 53 close contacts and people identified through enhanced surveillance, only one (Case 2) tested positive for influenza A(H5).

Active surveillance in the village identified six people with acute respiratory symptoms, and 19 people with respiratory symptoms presented to the mobile clinic for respiratory illness; all had respiratory specimens collected, and none tested positive for A(H5).

Investigations revealed that since early February, 22 chickens and three ducks died in the village. In early February 2023, wildfowl died at a lake ~50 km from Rumleach Commune; 9 of 29 of these tested positive for A(H5N1) [8]. Test results from samples of nine healthy chickens in the village taken on February 23 were negative for A(H5); testing results for samples taken on February 24 from two sick, one dead, and two healthy chickens, two ducks, and three environmental samples were not available from animal health authorities. By February 25, approximately 80% of an estimated 7500 chickens in the village had disappeared, likely sold or removed to avoid culling.

**FIGURE 1:**
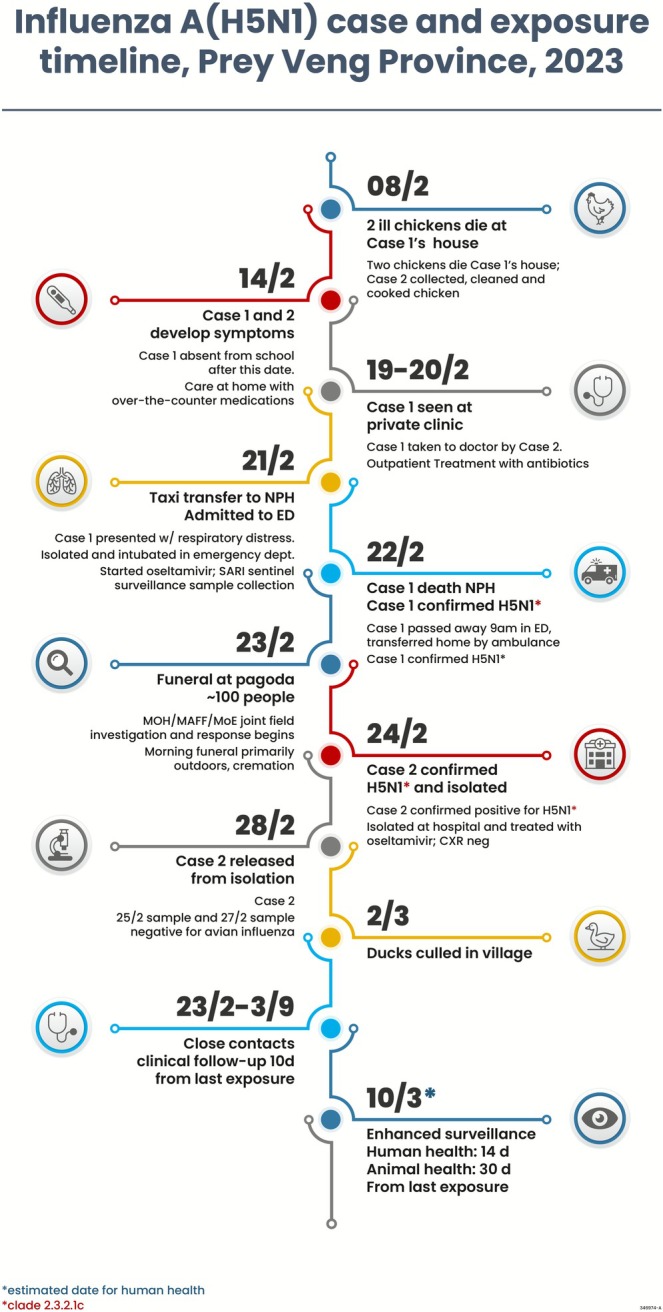
H5N1 case and exposure timeline.

On February 8, two chickens died at Case 1's house. Case 2 butchered the dead chickens, cooked, and ate them. Case 1 had exposure to the sick and dead chickens, which were not caged. The earliest known poultry exposures were before February 8 for Case 1 when the chickens first became ill and for Case 2 on February 8 when he butchered the dead chickens on February 8. Both Cases 1 and 2 had illness onset on February 14. Case 2 was exposed to Case 1 on February 14 when he took her to the clinic (Figure 1). The 6‐day incubation period after exposure to sick/dead poultry is long, but within the range of what has been previously reported [9, 10]. Neither case‐patient had recent exposure to ill persons before their illness onset. Paired sera were only available for Case 2, and serology did not identify HAI antibodies to A(H5N1) virus, but not all virologically confirmed cases that experienced mild respiratory illness have serologic evidence of a detectable antibody response [11]. The viruses isolated from the two human cases were virtually identical by sequencing and were the same clade 2.3.2.1e circulating among poultry in Cambodia at the time, and the cases were epidemiologically linked and had the same symptom onset dates; these findings strongly suggest that both cases most likely had the same exposure to sick/dead backyard poultry.

The limited A(H5N1) testing data in poultry and the environment is a gap in the investigation. Although waterfowl at a lake 50 km away from the cases' village died and tested positive for A(H5N1), poultry and environmental samples from the village tested negative or results were not available. However, for some laboratory‐confirmed A(H5N1) cases, the source is not always identified or the presumed exposure source did not have A(H5N1) testing confirmation [12]. Additional limitations include the lack of paired acute and convalescent serum specimens for some close contacts of both cases, and the use of serology only to detect HAI antibodies, but not neutralizing antibodies, to the A(H5N1) viruses isolated from both cases.

Investigators concluded that the source of infection for both cases was most likely from direct and close exposure to the same sick/dead backyard chickens and that human‐to‐human transmission was unlikely because the cases had the same illness onset date and did not have recent exposure to other sick contacts. Starting February 27, a series of health education seminars were conducted in the village on how to handle sick or dead poultry and what to do in case of respiratory illness. In response to the findings, animal health workers culled sick poultry in the village, starting on March 2.

## 4 Conclusions

A family cluster of confirmed cases of A(H5N1) virus, clade 2.3.2.1e, infection was identified in two blood‐related family members who had common exposure to sick/dead backyard poultry and the same date of symptom onset; one pediatric case developed pneumonia, respiratory failure, and died, and the other had mild illness. Clade 2.3.2.1e viruses circulating among poultry in the Mekong Delta pose a significant public health threat. Sixteen cases of clades 2.3.2.1e A(H5N1) virus infections were reported in Cambodia during 2023 and 2024, with six deaths [4].

## Author Contributions

**Savuth Chin:** methodology, investigation, supervision, project administration, writing – review and editing, data curation. **Chansovannara Soputhy:** investigation, supervision, project administration, writing – review and editing, data curation. **Heng Seng:** investigation, supervision, project administration, writing – review and editing. **Sokly Mom:** investigation, writing – review and editing, formal analysis. **Borann Sar:** conceptualization, methodology, investigation, formal analysis, writing – original draft, writing – review and editing. **Alyssa Finlay:** conceptualization, methodology, investigation, supervision, writing – review and editing. **Kathrine R. Tan:** conceptualization, methodology, investigation, formal analysis, supervision, project administration, writing – review and editing, writing – original draft, validation. **Philip L. Gould:** investigation, supervision, writing – review and editing. **Jurre Y. Siegers:** methodology, formal analysis, writing – review and editing, investigation, data curation. **Erik A. Karlsson:** methodology, investigation, formal analysis, supervision, writing – review and editing, data curation. **Sonja J. Olsen:** methodology, investigation, supervision, writing – review and editing. **Timothy M. Uyeki:** methodology, investigation, writing – review and editing. **William W. Davis:** methodology, conceptualization, investigation, formal analysis, supervision, writing – original draft, writing – review and editing, validation, data curation. **Darapheak Chau:** investigation, formal analysis, writing – review and editing. **Sovann Ly:** methodology, investigation, project administration, writing – review and editing, supervision.

## Funding

Over the past two decades, the US Centers for Disease Control and Prevention (CDC) has partnered with and supported capabilities in Cambodia to prepare and respond to emerging avian influenza threats for timely detection, response, and containment of this novel influenza virus that has global pandemic potential [13]. This activity was funded by a CDC Cooperative Agreement IP21‐2101 with the Cambodia National Institute of Public Health, with one of the objectives to “ensure capacity to respond to highly pathogenic viruses transmissible among humans.”

## Disclosure

The findings and conclusions in this report are those of the authors and do not necessarily represent the official position of the US Centers for Disease Control and Prevention.

## Conflicts of Interest

The authors declare no conflicts of interest.

## Supporting information


**Table S1:** Healthcare personnel exposures and PPE worn.

## Data Availability

The data that support the findings of this study are available from the corresponding author upon reasonable request.

## Endnotes

^1^
See, e.g., 45 C.F.R. part 46, 21 C.F.R. part 56; 42 U.S.C. §241(d); 5 U.S.C. §552a; 44 U.S.C. §3501 et seq.

^2^
(A/Cambodia/NPH230032/2023; GISAID: EPI_ISL_17024123).

^3^
(A/Cambodia/NPH2302009/2023; GISAID: EPI_ISL_17069010).
