# Supplementary material for: Investigation of a Family Cluster of Human Infections With Highly Pathogenic Avian Influenza A(H5N1) Virus, Clade 2.3.2.1e, in Cambodia, February 2023
Source: Influenza Other Respir Viruses. 2026 Feb 5;20(2):e70231. doi: 10.1111/irv.70231 (PMC12875686; doi:10.1111/irv.70231)
Supplement: Supplementary file 1 — Table S1: Healthcare personnel exposures and PPE worn. [file IRV-20-e70231-s001.docx]

| **Healthcare personnel** | **Clinic/Ward** | **Exposed to whom** | **Date of exposure** | **Exposure duration (min)** | **Distance <1m (Y/N)** | **Type of interaction** | **PPE worn** |  |
| --- | --- | --- | --- | --- | --- | --- | --- | --- |
| 1 | Romleach private clinic | Case 1, 2 | 17-Feb | 30 | Y | Consultation, examination, gave injection | None |  |
| 2 | Preay Deum Tleong private clinic | Case 1, 2 | 18-Feb | 30 | Y | Talk | Surgical facemask, cleaning hands with alcohol rub |  |
| 3 | Preay Deum Tleong private clinic | Case 1, 2 | 19-Feb | 30 | Y | Talk |  |  |
| 4 | Preay Deum Tleong private clinic | Case 1, 2 | 18-Feb | 30 | N | Talk |  |  |
| 5 | Preay Deum Tleong private clinic | Case 1. 2 | 19-Feb | 30 | N | Talk |  |  |
| 6 | Prey Sandek Market private clinic | Case 1, 2 | 19-Feb | 0 | N | Seen from distance | None |  |
| 7 | Prey Sandek Market private clinic | Case 1, 2 | 20-Feb | 0 | N | Seen from distance |  |  |
| 8 | Prey Sandek Market private clinic | Case 1, 2 | 19-Feb | 120 | Y | Touch, Talk | Surgical facemask, cleaning hands with alcohol rub |  |
| 9 | Prey Sandek Market private clinic | Case 1, 2 | 20-Feb | 120 | Y | Touch, Talk |  |  |
| 10 | NPH | Case 1 | 21-Feb | >120 | Y | Intubation | PPE level 2 |  |
| 11 | NPH | Case 1 | 22-Feb | >60 | Y | Suction | PPE level 2 |  |
| 12 | NPH | Case 1 | 21-Feb | 60 | Y | Intubation, touch | Surgical facemask, gloves |  |
| 13 | NPH | Case 1 | 22-Feb | 30 | Y | Aspiration, touch | Surgical facemask , gloves |  |
| 14 | NPH | Case 1, 2 | 21-Feb | Multiple exp over 24h | Y | Touch, talk | None |  |
| 15 | NPH | Case 1 | 21-Feb | 90 | Y | Touch, talk | Surgical facemask, gloves |  |
| 16 | NPH | Case 1 | 21-Feb | 90 | Y | Touch, talk | Surgical facemask, glove |  |
| 17 | NPH | Case 1 | 21-Feb | 90 | Y | Touch, talk | Facemask, glove |  |
| 18 | NPH | Case 1 | 21-Feb | 30 | Y | Touch, talk | Mask, glove |  |
| 19 | NPH | Case 1 | 21-Feb | 300 | Y | Touch, talk | Mask, glove |  |
| 20 | NPH | Case 1,2 | 21-Feb | 300 | Y | Touch, talk | Surgical facemask, glove |  |
| 21 | NPH | Case 2 | 21-Feb | 5 | N | Talk | Surgical facemask, glove |  |
| 22 | NPH | Case 1 | 21-Feb | Multiple exp over 24h | Y | Touch, talk | Surgical facemask, glove |  |
| 23 | NPH | Case 2 | 21-Feb | Multiple exp over 24h | N | Touch, talk | Surgical facemask, glove |  |
| 24 | NPH | Case 1 | 21-Feb | Multiple exp over 24h | Y | Touch, talk | Surgical facemask, glove |  |
| 25 | NPH | Case 2 | 21-Feb | Multiple exp over 24h | N | Talk | Surgical facemask, glove |  |
| 26 | NPH | Case 1 | 22-Feb | 90 | Y | Touch, clean body | PPE level 2 |  |
| 27 | NPH | Case 1 | 21-Feb | 90 | Y | Touch, talk | PPE level 2 |  |
| 28 | NPH | Case 1 | 22-Feb | 30 | Y | Touch, talk | PPE level 2 |  |
| 29 | NPH | Case 1 | 22-Feb | 90 | Y | Touch, clean body | PPE level 2 |  |
| 30 | NPH | Case 1 | 22-Feb | 15 | Y | CPR, suction | PPE level 2 |  |
| 31 | NPH | Case 1 | 21-Feb | Multiple exp over 24h | Y | Touch, clean | Surgical facemask, glove |  |
| 32 | NPH | Case 1 | 21-Feb | Multiple exp over 24h | Y | Talk | Surgical facemask, glove |  |
| 33 | NPH | Case 1 | 22-Feb | 60 | Y | CPR, suction | Surgical facemask |  |
| 34 | NPH | Case 1 | 22-Feb | 60 | Y | Suction, blood draw | Surgical facemask |  |
| 35 | NPH | Case 1 | 21-Feb | 120 | Y | Touch, clean body, talk | Surgical facemask, gloves |  |
| 36 | NPH | Case 1 | 21-Feb | 120 | Y | Mechanical ventilation | Surgical facemask |  |
| 37 | NPH | Case 1 | 22-Feb | 15 | Y | Physical examination | Surgical facemask |  |
| NR = not reported; NPH = National Pediatric Hospital; PPE level 2 is gown, facemask, safety glasses, face shield, shoe covers, gloves | | | | | | | | |
